# Physician leadership in private acquisitions: Styles for all situations

**DOI:** 10.1016/j.clinsp.2024.100443

**Published:** 2024-07-18

**Authors:** Max S. Mano, Fadil Çitaku

**Affiliations:** Academy of Leadership Sciences Switzerland, Zürich, Switzerland

Within the range of skills that are expected from a medical leader, leadership style matters more than many. The most relevant leadership styles that modern medical leaders should be acquainted with and their relationship with the sources of power are summarized in Table 1. However, as medical practice and employment change and grow in complexity, leaders must develop the skills and awareness to switch from one style to another, according to the situation.

**Table 1:** Sources of power for a medical leaders and compatible leadership styles.

Sources of power			Compatible leadership styles
Positional power	Reward power	Follower`s perception that the leader can mediate reward to him	Transactional Leadership: in this style, the leader is perceived as having the ability to mediate rewards, indicating a transactional exchange between the leader and followers based on performance and outcomes
	Coercive power	Follower`s perception that the leader can mediate punishment to him (e.g., be fired)	Coercive Leadership: coercive leaders rely on the use of threats or punishment to influence followers. This style can be effective in situations where immediate compliance is necessary. In this case, the leader's ability to mediate punishment aligns with the coercive leadership style
	Legitimate power	Follower`s perception that the leader has a legitimate right to prescribe a behavior for him because of a status or job authority	Authoritarian Leadership: in this style, the leader makes decisions independently and expects followers to comply without much input or discussion. The focus is on clear hierarchy and obedience to authority
Personal power	Referent power	Based on follower`s identification and liking of the leader	Transformational leadership: transformational leaders inspire and motivate their followers by creating a strong sense of identification and admiration. They appeal to followers' emotions, values, and aspirations, fostering a positive and supportive relationship
	Expert power	Based on follower`s perception of the leader`s competence knowledge	Transformational
			Charismatic
			Path-go
			Leader-member exchange
			Team leadership
	Neuroleadership	Neuroleadership is a leadership approach that integrates findings from neuroscience into leadership practices. It emphasizes understanding how the brain functions and apply that knowledge to enhance leadership effectiveness.	There isn't a specific leadership style associated with neuroleadership; instead, it involves incorporating insights from neuroscience into various leadership styles
	Emotional Intelligence Leadership	Emotional Intelligence refers to the ability to recognize, understand, and manage one's own emotions, as well as to perceive and navigate the emotions of others effectively	Emotional Intelligence is essential for effective leadership across various styles and is a prerequisite for optimal leadership in numerous situations

Perhaps the greatest single change in medical practice is the tendency in many countries for physicians to become less independent as their private practices, hospitals and healthcare microsystems are acquired by big private equity companies – meaning that they will need to learn new skills to adapt and, why not, even thrive in the heart of the corporate world.

Firstly, when this decision happens to be in their hands, physicians should understand the full meaning of selling out their practices – such as immediately losing control over its management, their own leadership, and, most likely, having many people hierarchically above them. There's also the (recently mediatized) risk of quality deterioration in the aftermath of an acquisition – another potential source of distress.^1^ It's also worth noting these companies’ strong drive to develop big data infrastructures which is apparently required to make the business thrive; however, because physicians are often the primary source of data, they can easily fall prey to the “Electronic Health Records (EHR) trap” that is at the heart of the 21st century's burnout crisis among physicians.

Following an acquisition, physicians may become bound by a contract that ensures their continued services for the company for some time. However, bounding contracts (especially when protected by cancellation penalties) may provide new company leaders *position power* that entitles them to use reward/coercive techniques, in other words, allowing them to more easily employ leadership styles that focus on rewards (i.e., incentives) and/or punishments. Yet, recent studies have precisely pinpointed the nature of the incentives as the main cause of quality deterioration in the aftermath of an acquisition.^1^

Of note, coercive/punishment strategies are unlikely to work for more conventional (unprotected by cancellation penalties) employment contracts, because unsatisfied physicians can simply leave or even ignore intolerable new rules – this way staying out of the range of power of the leader. Whether or not a coercive/punishment leadership style ever makes sense when dealing with highly educated people, physicians should be aware that certain contract terms can make them vulnerable to this strategy.^2^

In conventional employment contracts, the leader may still retain some legitimate power, provided the followers also have this perception; the possibility of contract termination is an example of residual power. However, this will work only within certain limits and is unlikely to allow the employment of a classic coercive/reward leadership style. In this situation, the leader is expected to use referent power (which is based on the followers’ identification and liking of the leader) – which has a broader range of power, as well as expert power (which is based on followers’ perception of the leader`s competence or knowledge) and adopt a leadership style that is compatible with these sources of power (Table 1). Usually, considering that physicians are highly educated and trained, and especially when working in a well-structured institution, they will only need a supportive type of leadership, which employs two-way communication more in the sense of providing social and/or emotional support.

For even more fragile contract bonds, for instance, a group of physicians independently working under the same roof (as “third-party service providers” – now a dominant model in the first author's reality), there can still be room for influence of referent power, but leadership will be mainly exercised based on expert power. Both are considered personal sources of power, instead of position. This means that the leader will need to have the skills to emanate trust, which is the basis for sustaining this source of power. One difficulty here is the fact that the range of power is more limited and may be restricted to the leader's area of expertise. Leadership styles compatible with this scenario include transformational or charismatic, path-go, and leader-member exchange (Table 1).

Another challenging scenario for medical leaders is the growing tendency of insurance companies to withdraw from contracts with service providers to become providers themselves – one of the most controversial aspects of a wider process known as “vertical integration”. This process has several implications, for instance, from the standpoint of insurance providers, in terms of reducing costs, litigation risks, and tackling competition – this way promoting financial sustainability for the business; however, from the standpoint of clients and physicians, it can be seen in many ways as a potential conflict of interest, such as in terms of harming the healthy convention of diverging interests between parties. From the leadership perspective, in a verticalized system, physicians – holders of the power over prescriptions which are considered major drivers of costs – become true employees and this way more tightly attached to institutional (i.e., insurance companies) treatment protocols, because they can now be punished/rewarded according to their adherence to these protocols. This has been particularly problematic in places where there is no consensus on who holds the authority to rule treatment guidelines. Addressing the controversies about the efficiency, ethics, and legality behind verticalization in healthcare is beyond the scope of this piece;^3^ however, it represents perhaps the greatest single change in terms of physicians becoming subject to leadership *position power*, raising concerns about further pressures being put on them. Physicians aspiring to leadership positions under these circumstances should be aware of the challenges ahead, especially in terms of potential ethical dilemmas and conflicts with peers.

In challenging times, effective leadership becomes a must. Well-prepared leaders must be able to safely navigate through changes and provide much-needed support for their peers – while, at the same time, watching over the sustainability of the system and safeguarding quality of care. For this, they must be aware of the rapidly changing scenario and learn to adjust their actions according to each situation.

## Declaration of competing interest

The authors declare no conflicts of interest.
